# Letter from J. P. and J. H.

**Published:** 1890-10

**Authors:** 


### Correspondence.

Mr. Editor :—Your "leaders" to the Apostolatous letter of Dr. Hayd in your August number were very fitting, and a real fore-shadowing of what was to come. In fact, when a true follower of the prophet journeys to Mecca, we know beforehand that a new manifesto is to be promulgated, in which Mahomet is deified and the Koran glorified, where faith shines refulgent as a knowledge of things hoped for, an evidence of things unseen.

This is especially applicable to the electrical diagnosis which is charmingly and alluringly exact to the unstudied. In electricity, as in other magic, success is dependent upon its factors, faith, paraphernalia, and plausibility, which stands for honesty ; not maintaining, however, that there are no honest electricians. It is noteworthy, nevertheless, that such men, honest because surgically capable of estimating the claims of electricity by reason of experience, are foremost in narrowing its usefulness. Or, to state the proposition more directly : to make good a claim for any remedy, as a specific, which electricity is claimed to be in the diseases of women, it is necessary for those so asserting to know these diseases, their lesions, their diagnosis and treatment, their complications and pathology, to an extent which will justify their claim to be considered authorities concerning them. To illustrate : Dr. Apostoli, when advocating electrolytic galvanic puncture in extra-uterine pregnancy, was asked whether he had ever used it. His answer was "no." The inference is, then, that if he had never used it he had never seen a case, and if he had never seen a case he was thoroughly incompetent to say what should or should not be the treatment. His opinion may have been very honest, but the expression of it, as an authority, was dishonest. A man may cast counterfeit coin, but he has no right to stamp it as genuine and pass it. Now, we hold such is the position of most electricians ; they substitute for experience and knowledge, which would teach them in a majority of cases the impossibility of exact diagnosis to start with, a presumptuous pretense at infallibility in diagnosis. They create the disease, then cure it ; or, finding a physiological variation, they juggle with pathological terms until the two are confounded, and the general reader is confused. Such, for instance, is their use of the term "fibroid uterus" as against a fibroid tumor of the uterus. Such, again, is their position as to the frequency of fibroid tumors necessitating active interference. Says the writer: "One sees a great many fibroids of all sizes and in various locations; some are extremely large and have been under observation for many years." As a matter of fact, fibroids requiring radical treatment, speaking out of the knowledge afforded by a large clinic, are not numerous. But granting they are numerous, why, if electricity cures them symptomatically, as is claimed, are they kept under observation "for many years?" "In exceptional cases the tumor has disappeared," is one of the pleasant fables (Credat Judæus Apelles) given out to draw, Louisiana lottery fashion, but will never stand good save for the phantoms of hysteria.

We fear his evident desire to be pleased has led him to undue enthusiasm at a sacrifice of his better judgment, else how were it possible for him to say in all fairness "he (Apostoli) is a brilliant diagnostician?" Is he not too easily satisfied? Said the guide: "This is the tomb of Adam, for it has never been proved to the contrary." And here we must insist, for his enlightenment and for the benefit of those who may be misled by his enthusiasm, that absolute or even approximate diagnosis of the conditions likely to be found in relation with uterine fibroids, worthy of the name, is impossible even by those who are accustomed to deal with these tumors visually, hand to hand, or even stage to stage, in operation. The claims made by these men are wild, and would be ridiculous were they not fraught with such a fearful burden of falsehood. If the author of the letter considers such expression extreme, we can afford him opportunity, to his satisfaction, to prove or disprove the fairness of our position practically.

The writer champions Dr. Apostoli; this is just, but ill-advised. He says: "What his earlier utterances and claims may have been, I know not, but the position which he now takes in reference to electro-therapy, as applied to diseases of women, is not as extravagant as we in America have supposed." The näivete of all this is charming, but fatal to the former extravagances claimed by him, as expressed in Buffalo in April last, when he followed, defended and extolled the Apostoli treatment, all without knowledge of it, crying like the priests before the sacred shrine, "*procul, procul, este nefandi!*" to those who criticized and discredited the pretentious claims of the man, because from experience and study they had a right so to do.

As to the extent to which Apostoli claims the usefulness of electricity in diseases of women, we have his sanction in Bigelow's compilation, 1889, to which he stands godfather for the following: Fibroid Tumors of the Uterus, Hypertrophy of the Uterus, Non-Suppurative Salpingitis, Metritis, Endometritis, Subinvolution, Superinvolution, Disorders of Menstruation, Ovarian Pain, Chronic Oöphoritis, Peri-uterine Inflammations, Displacements, Hematocele, Some Hystero-neuroses, Stenosis of the Cervical Canal, Erosions of the Cervix, Nausea of Pregnancy — all of which would justify the writer's exclusive use of electricity in all complaints of women, as expressed in his Buffalo discussion in April last, and show if it is not now in accord with the teachings of his master, that his electrical therapeutics are about as variable as many of the physical manifestations of the subtle force, and no more certain of permanency.

As to the cure of uterine fibroids, he is strangely "conservative" as to the efficacy of electro-puncture. We at first feared another Apostolical dogma had failed of its infallibility! But, now that we know "puncture" means only a *prick*, varying in depth from one-twentieth to one-twelfth of an inch, we are at peace, and must war no more against it as being dangerous, or having the elements of being worthy of serious consideration. He is also sure of "cellulitis." We adjure him, has he ever, to his positive knowledge, or in post-mortem examination, seen a case of simple cellulitis? Will he take the testimony of those who have looked for it and have failed to find it? Will he read the modern pathology, now almost ancient, apropos of Bernutz and Goupil, and alone convince himself that together with his own ignorance of the "earlier" views of his master, and the master's own ignorance or disregard of all that is proven by direct investigation in pelvic disease, that cellulitis, generally speaking, is a myth, and, therefore, that its cure is mythical?

As to the pain in the application of electricity, "They jest at scars who never felt a wound," and it needs only the testimony of women who have felt the pain, to convince all but the electromaniacs that there is pain, and this even is confessed by them in their rules for the application of the current.

So far as the cure of hemorrhage is concerned, we must, first of all, understand clearly what "cure" means. There is no doubt that the cauterizing, or cooking, effect of the galvanic current is temporarily hemostatic in its effects, but that one, or two, or more applications will permanently cure uterine hemorrhage, without continual recourse to the current, has not been proven. On the contrary, there are numerous instances in which the hemorrhage has been rendered much worse or incontrollable after the application of the current. But even here he is self-contradicting, for further on he says: "I have seen cases come to the clinic who bled profusely after an examination. This treatment lasted several minutes with the effect of completely arresting hemorrhage for several days." Altogether there is little to convince, and much to discourage, faith in electrical methods as expounded in his correspondence.

The writer's sublime confidence equals that of the poet who declares of his mistress: "I do believe her, though I know she lies." After all, infatuation is not reason, nor is asseveration argument. More light is needed to clear away the conflicting statements and opinions concerning this gynecological panacea, as held by the electricians, or this most arrant of frauds, as believed by the surgeons. J. P. and J. H.

---

### COURT RECEPTION AT BERLIN.

By command of the Emperor, a Reception and Garden Concert was given at Potsdam, at which Prince Leopold, of Prussia, the Emperor's cousin, received the guests, attended by Count Caprivi, Court Marshal Count Eulenberg, Herr von Gossler, (Minister of Education,) and other high officials. Invitations were issued to 500 guests, selected from many of the leading British delegates and members. At the reception, which took place in the Shell Room, the guests were arranged according to their nationalities, and Prince Leopold, in walking round the circle, caused a number of the representative visitors to be specially presented to him, addressing to each of them a few words of courteous welcome. Subsequently a concert was held in the garden, in the course of which Prince Leopold mixed with the visitors, and conversed with them on subjects likely to interest each. Refreshments were served, and the party returned, being taken back to Berlin by the special train which had brought them. Everything was done to make the medical guests of Germany feel that the German Emperor fully participated in the cordial and generous welcome which had been given to them by the municipality, citizens, and the profession of Berlin, and desired to mark his gracious goodwill to the Congress and its members. Happily, the weather on this as on all the other days of the Congress was peculiarly favorable, and the Court function was in every way an interesting and successful one.—*Journal American Medical Association.*

